# Distinct profile of cell-free DNA in malignant pleural effusion of non-small cell lung cancer and its impact on clinical genetic testing

**DOI:** 10.7150/ijms.52306

**Published:** 2021-01-30

**Authors:** Yongfeng Yu, Jie Qian, Lan Shen, Wenxiang Ji, Shun Lu

**Affiliations:** Shanghai Lung Cancer Center, Shanghai Chest Hospital, Shanghai Jiao Tong University, Shanghai, China.

**Keywords:** lung cancer, pleural effusion, cell-free DNA, long fragment, genetic analysis

## Abstract

Cell-free DNA (cfDNA) in supernatant of pleural effusion from advanced NSCLC patients has been proved as surrogate sample detecting therapeutic targets as well as tumor mutation burden (TMB). As recently reported, cfDNA in pleural effusion supernatant is superior to plasma in TMB evaluation. It is reasonable to hypothesize that cfDNA profile in pleural effusion (PE) and plasma might be different. It remains to be elucidated why cfDNA in PE supernatant impacts on genetic analysis. Consequently, the approach dealing with cfDNA from PE supernatant might need to be different from that for plasma cfDNA in order to obtain accurate clinical genetic testing result.

**Methods:** Pleural effusion samples from 32 patients with stage IV lung adenocarcinoma were collected. Supernatant and sediment were processed separately to extract Cell-free DNA as well as sediment DNA (PE-S). cfDNA from pleural effusion was analyzed by Agilent 2100 bioanalyzer. Libraries were prepared by 1) direct use of the total cfDNA without fragmentation step (PE-FL) or 2) use of full-length cfDNA fragmented to 150-250bp (PE-F), 3) use of cfDNA fragments enriched to ~167bp (PE-E167) as well as 4) use of cfDNA fragments larger than 500bp enriched (PE-E500). All samples were subjected to targeted next-generation sequencing (NGS) with a panel of 448 cancer-related genes as well as a panel of 10 NSCLC driver genes.

**Results:** cfDNA were successfully extracted from 30 MPE samples. cfDNA displayed distinct profile in supernatant of malignant pleural effusion from that of plasma cfDNA. No statistical difference in detection of hotspot variations between PE-E167 and PE-F by 448-gene or 10-gene panel. While TMB from PE-F samples was significantly higher than that from PE-E167 and PE-FL. Higher TMB from PE-F was resulted from cancer-unspecific variants with low allele frequency (0.1%-1%) which were mainly introduced by long-fragment cfDNA. Similar genetic profile was observed between paired cfDNA of PE-FL and cfDNA of PE-E167.

**Conclusion:** Long-fragment cfDNA in the PE supernatant will introduce low abundant cancer unrelated variants which leads overestimation of TMB. Paired PE-FL and PE-E167 gave comparable outcomes. Direct use of the total cfDNA without fragmentation step (PE-FL) is recommended for library preparation of NGS testing in clinical practice to exclude interference from long fragments of the cfDNA.

## Introduction

To take advantage of targeted therapy and immune checkpoint inhibitors (ICIs), molecular profiling is pre-requested 1-3. In clinical practice, it is not always available to obtain sufficient tumor tissue for molecular profiling, especially from patients with advanced tumors. Thus, liquid biopsy, as an approach more feasible to assess circulating tumor DNA (ctDNA) by using blood or other body fluid samples like sputum 4 and malignant pleural effusion 5, has been proved to guide targeted therapy as well as predict efficacy of immunotherapy by yielding trustable results of driver gene mutations as well as bTMB respectively 6.

Malignant pleural effusion (MPE), a common complication of lung cancer, defined as excessed fluid accumulated between lung and pleural cavity, is significantly associated with patients' poor prognosis. Drainage of excessive MPE provides a good opportunity to obtain sufficient source to assess tumor genomics 7. Sedimental cell pellet has been used for cytological diagnosis as well as genetic testing 8. While, due to the complexity of cell compositions, especially in hemorrhagic PEs 9, large numbers of erythrocytes as well as leukocytes, would largely impact the sensitivity of detecting somatic mutation. Cell-free DNA (cfDNA) in the supernatant of MPE has been proved to be superior to sedimental cell pelleting in detection of genetic variants as well as tumor mutation burden (TMB). Moreover, as Tong et al. recently showed, tumor-derived DNA from PE supernatant can be a better sample source than plasma cfDNA for assessing genetic aberrations as well as TMB in patients with advanced NSCLC when tumor tissue is not available 10. In addition to higher DNA concentration in MPE giving rise to higher TMB evaluation, differential cfDNA fragment profile in MPE could be a putative factor making genetic testing different. While, by far, cfDNA profile in MPE was not thoroughly investigated.

**Figure 1:**
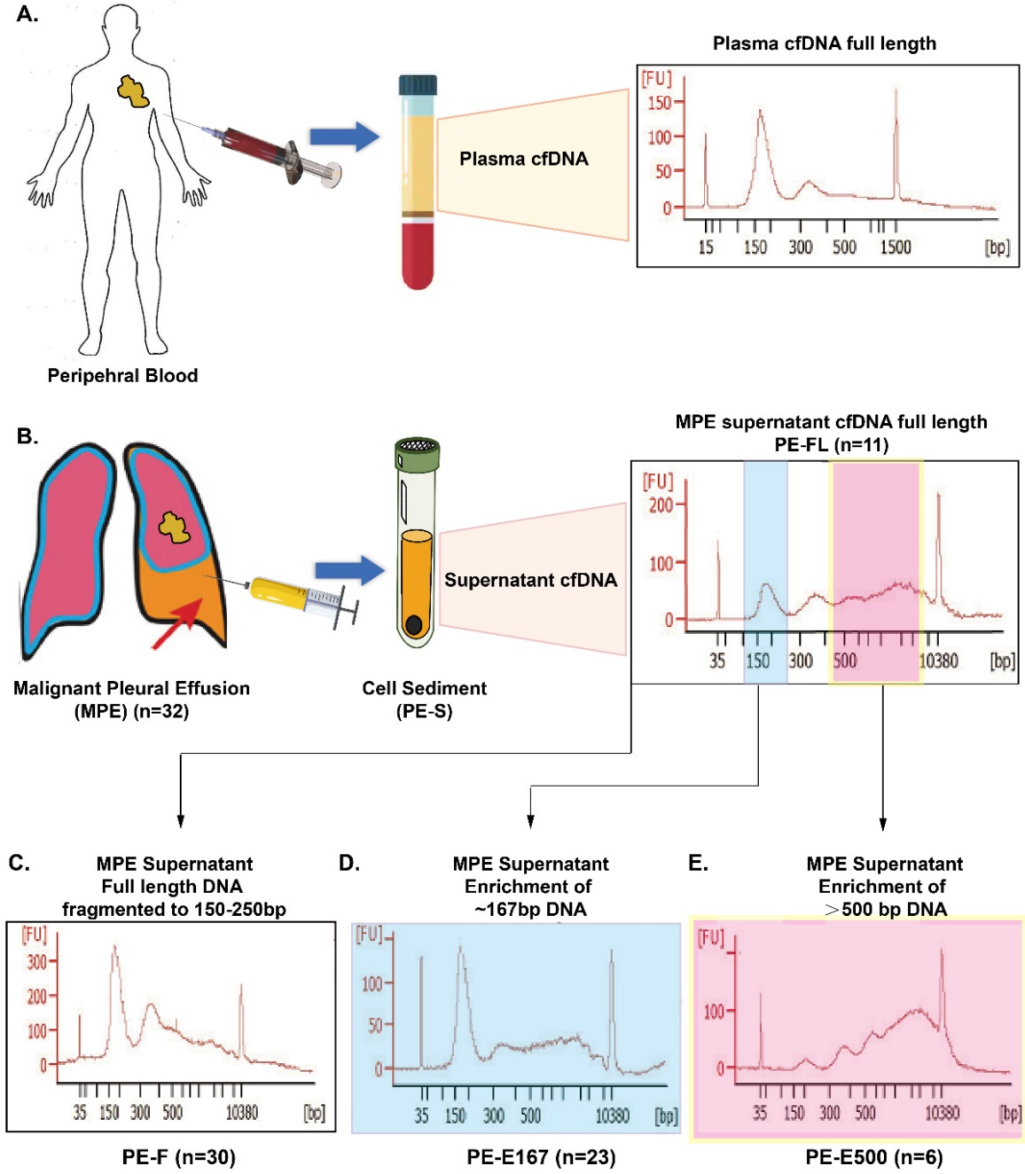
** Distinct cfDNA profile in Malignant pleural effusion. A.** Cell-free DNA (cfDNA) distribution in plasma as reference. **B.** Distribution of full length cfDNA in malignant pleural effusion (PE-FL). **C.** cfDNA in malignant pleural effusion fragmented to a size range from 150 bp to 250 bp (PE-F). **D.** Enriched 167 bp cfDNA fragment from malignant pleural effusion. **E.** Enriched cfDNA fragment larger than 500 bp.

Fragment size of cfDNA is heterogeneous in multiple types of body fluid 11. In plasma, peak cfDNA fragments are approximately 167 bp, accompanied by sizes in multiples of 167 bp due to apoptosis induced nucleosome releasing. While long-fragment cfDNA, with size of more than 10,000 bp, was reported by previous study on plasma cfDNA samples 12. Our preliminary data displayed that, compared with plasma, cfDNA in supernatant of MPE contains much more longer DNA fragments with size of longer than 500 bp (Figure 1A, 1B). However, whether those longer cfDNA fragments in PE supernatant will have impact on genetic testing, and how to deal with these longer fragments in clinics is still unclear.

In this study, cfDNA in supernatant of MPE was extracted followed by comprehensive analysis of driver mutations as well as TMB by NGS applying 10-gene and 448-gene panel respectively. Fragmented full-length cfDNA (PE-F, n=30) in supernatant of MPE which contained entire genetic information was prepared. Further, the impact of PE-F on genetic analysis was thoroughly investigated by comparing tested results with PE-FL (direct library preparation from the total cfDNA without fragmentation step) or PF-E167 (enriched 167 bp fragments). To clarify the influence of long-fragment cfDNA on detecting genetic aberrations, differences of genetic aberrations between long-fragment cfDNA and PE-E167 or PE-FL were investigated.

## Materials and Methods

### Patient recruitment and sample collection

From May 2018 to February 2019, 32 patients with stage IV, pathologically confirmed lung adenocarcinoma were enrolled. MPE were drained and collected due to relieving clinical symptoms at diagnosis or during disease progression at Shanghai Chest Hospital of Shanghai Jiao Tong University. Clinicopathological characteristics and survival data of 32 patients with lung cancer were obtained and combined with high-throughput sequencing data for joint analysis. Patients' baseline data are provided in Table 1. This study was approved by the ethics committee of Shanghai Chest Hospital of Shanghai Jiao Tong University, and all patients provided informed written consent. All samples were tested at a clinical genomics testing laboratory (AmoyDx, Xiamen, China) using protocols approved by the ethics committee of Shanghai Chest Hospital of Shanghai Jiao Tong University.

MPE were collected simultaneously from all 32 patients. Fifty milliliters of MPE was collected into a 50 ml sterile centrifuge tube (Corning, NY, USA) and centrifuged at 3000g for 10 min at room temperature to separate the PE supernatant and PE cell sediment, which were subsequently used for cfDNA and genomic DNA (gDNA) extraction, respectively. Then, 2-4 ml of anticoagulated whole blood was collected in EDTA anticoagulation tubes (BD, CAT, USA), and centrifuged at 1600 g for 10 min at room temperature to isolate white blood cells for gDNA extraction and as a germline control.

### DNA extraction, enrichment, and library construction

CfDNA was extracted from PE supernatant using the QIAamp Circulating Nucleic Acid Kit (Qiagen, Hilden, Germany) following the standard protocol. gDNA from PE cell sediment and white blood cells were extracted using the AmoyDx Blood and Leukocyte DNA Kit (AmoyDx, Xiamen, China). All DNA was quantified using the QuantFluor® dsDNA System on a Quantus™ Fluorometer (Promega, USA). CfDNA from thirty samples were successfully extracted. Fragment distribution was analyzed in a Bioanalyzer 2100 using the High Sensitivity DNA Kit (Agilent Technologies, Santa Clara, CA). Full-length cfDNA from PE supernatant without fragmentation step during NGS library preparation was termed PE-full-length (PE-FL, Figure 1B) (11 of 30 samples). To obtain all information from cfDNA in PE supernatant, full length cfDNA was fragmented to a size range from 150 bp to 250 bp using Covaris M220 (Covaris, Woburn, MA) (terminated as PE-F, 30 samples, Figure 1C). CfDNA from PE supernatant was separated based on size using Agencourt Ampure XP beads (Beckman Coulter, Indianapolis, IN). AMPure XP beads in 0.6× and 1.2× and 0.6× volumes were used to enrich DNA ~167 bp (PE-Enrichment 167 [terminated as PE-E167, 23 of 30 samples], Figure 1D) and > 500 bp (PE-enrichment 500 [PE-E500], 6 of 30 samples, Figure 1E), respectively. DNA from paired PE cell sediment (PE-Sediments [terminated as PE-S], 30 samples) were fragmented to a size range from 150 bp to 250 bp using Covaris M220 (Covaris, Woburn, MA). All DNA fragments were analyzed in a Bioanalyzer 2100 using the High Sensitivity DNA Kit (Agilent Technologies, Santa Clara, CA). The sequencing library was constructed using the NEBNext Ultra II DNA Library Prep Kit for Illumina (New England BioLabs, Beverly, MA) using the standard protocol. Pre-capture libraries were established after end-repairing, A-tailing, adaptor ligation and PCR with indexed primers, all following the manufacturer's recommendations for probe hybridization and targeted capture with the AmoyDx pan-cancer 448-gene panel (Table S1) and lung cancer 10-gene panel (Table S2). Captured libraries were purified and amplified to obtain the post-capture library. Final sequencing of the library was obtained through quality assessment and using quantitative real-time polymerase chain reaction (PCR).

### Sequencing and data processing

All normalized libraries were pooled, and DNA was sequenced in an Illumina NovaSeq 6000 platform with 2 × 150-bp pair-end reads. Sequencing data were first cleaned to remove sequencing adaptors and low-quality reads (quality < 15) or poly-N with Trimmomatic and mapped to human reference genome, version 19 (hg19) using the Burrows-Wheeler Aligner. PCR duplicates were marked and removed using Mark Duplicates from the Genome Analysis Toolkit (GATK). Base Quality Score Recalibration was performed using GATK's BaseRecalibrator and ApplyBQSR. After correction, a bam file was acquired. The InDels and single-nucleotide polymorphisms were compared by Mutect2 and FilterMutectCalls of GATK to obtain the final vcf file.

For analyzing hotspot mutations, the variant allele frequency (VAF) cutoff was 0.1% for both 10-gene and 448-gene panel 10. ANOVA was used to annotate the vcf file into a MAF file to filter out common mutations and germline mutations. The MAF file was used for subsequent data analysis.

Referred to the algorithm of plasma bTMB in 2018 13, the TMB of PE samples was determined by identifying all SNVs at an VAF either ≥ 0.5% or 0.1% as proposed by Tong et,al 10 across the coding region of 448 genes (~1.4 Mb) and filtering out germline mutations by paired blood samples. In addition, the 1000 Genomes and gnomAD database were compared to remove germline mutations with a frequency greater than 1% in healthy individuals.

### Statistical analyses

The paired Student's t test was applied for continuous variables in box plot and violin plot. All statistical analyses were performed using SPSS 23.0 (IBM, Armonk, NY) and R (version 4.0.1; The R Foundation, Vienna, Austria; http://www.R-project.org).

## Results

### Patient baseline characteristics

This study enrolled 32 patients with stage IV lung adenocarcinoma who had MPE at diagnosis or during disease progression (Table 1). The median age at enrollment was 58 years (range 43-77). 17 patients (60.7%) were smokers. Smoking history of four patients was not available. More than half of the patients were male (21, 65.6%), and 10 (31.2%) had *EGFR* mutations. 15 patients at clinical stage IVA (M1a stage, 25%; M1b stage, 21.9%) and 17 at clinical stage IVB (M1c stage, 53.1%). All patients were pathologically confirmed adenocarcinoma.

### Cell-free DNA in malignant pleural effusion displayed distinct profile

Among 32 samples, cell-free DNA were successfully extracted from 30 samples with accepted quality. DNA fragments were analyzed via Bioanalyzer 2100 yielding a distinct cfDNA distribution in PE supernatant, especially containing longer DNA fragments which had not been observed in plasma cfDNA (Figure 1A, 1B). To verify whether the long DNA fragments in the MPE supernatant will affect genetic test results including driver mutations as well as tumor mutation burden, full-length cfDNA from PE supernatant (PE-F) were fragmented to sizes ranging from 150 bp to 250 bp (Figure 1C) assuming including entire genetic information in MPE, followed by being tested via a 10-gene and a 448-gene panel respectively. The results were compared with those from either PE-E167 or PE-FL as well as PE-E500 (Figure 1B, 1D, and 1E). Meanwhile, genetic profiling was performed simultaneously for whole set of PE samples (PE-F, PE-E167, PE-E500 and PE-FL) from 4 patients.

### Fragmented full-length cfDNA (PE-F) displayed comparable hot-spot driver mutations with PE-FL or PE-E167

**Figure 2:**
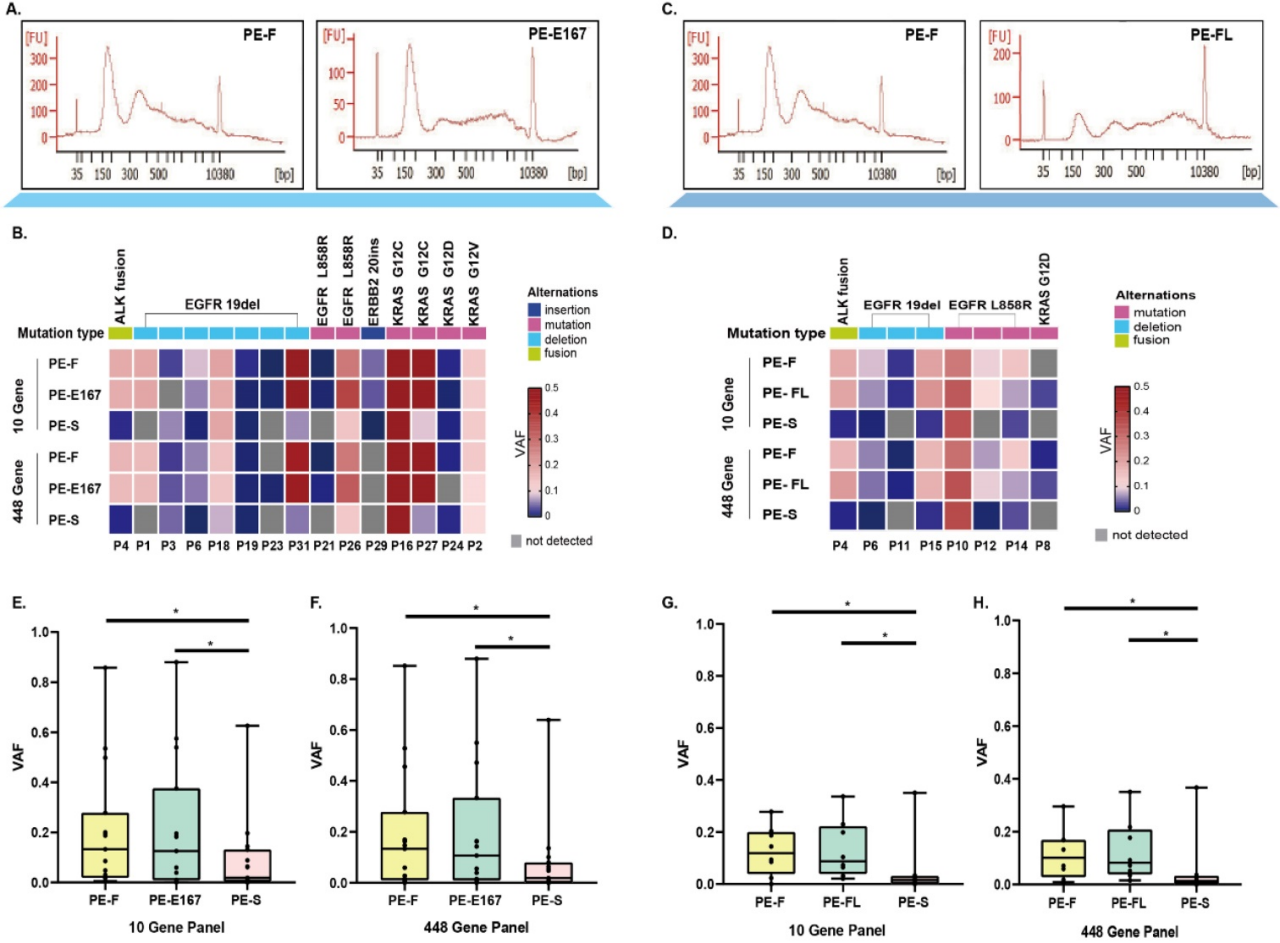
** Comparing hotspot variants and tumor mutation burden among PE-E167, PE-FL, PE-F and PE-S. A.** Comparing hotspot result between PE-F and PE-E167 samples. **B.** Hotspot variants detection rate of driver genes in PE-F, PF-E167 and PE-S using an NGS 10-gene and 448-gene panel. Variation types and VAF are indicated by different colors. **C.** Comparing hotspot result between PE-F and PE-FL samples. **D.** Hotspot variants detection rate of driver genes in PE-F, PF-FL and PE-S using an NGS 10-gene and 448-gene panel. Variation types and VAF are indicated by different colors. **E.** VAF of PE-F and PE-E167 were significantly higher than that of PE-S in NGS 10-gen panel, * p < 0.05. **F.** VAF of PE-F and PE-E167 were significantly higher than that of PE-S in NGS 448-gen panel, * p < 0.05. **G.** VAF of PE-F and PE-FL were significantly higher than that of PE-S in NGS 10-gene panel, * p < 0.05. **H.** VAF of PE-F and PE-FL were significantly higher than that of PE-S in NGS 448-gene panel, * p < 0.05.

No significant difference of hot spot driver mutations was found between two comparison categories (Figure 2A and 2B for PE-F vs PE-E167, Figure 2C and 2D for PE-F vs PE-FL) no matter via 10-gene or 448-gene panels. Further, variant allele frequency (VAF) of mutations in PE-F has no difference compared with PE-E167 or PE-FL (Figure 2E, 2H). More hotspot variations with statistical significance were detected in both of PE-F, PE-E167 or PE-FL than paired PE-S (Figure 2E, 2H, p < 0.05, respectively).

### Fragmented full-length cfDNA (PE-F) displayed unusual TMB distribution

**Figure 3:**
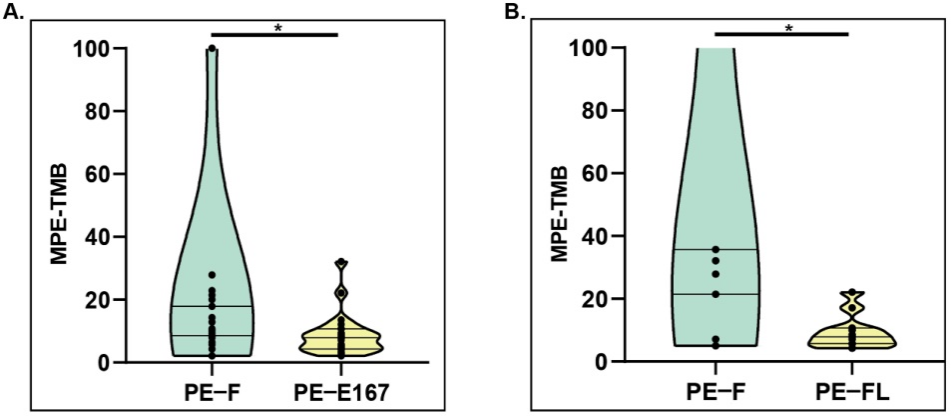
** Comparing TMB distribution among various sample types. A.** PE-TMB was calculated using the plasma bTMB algorithm. The TMB calculated from PE-F were significantly higher than that calculated from PE-E167, *p < 0.05. **B.** The TMB calculated from PE-F were significantly higher than that calculated from PE-FL, *p < 0.05.

Validated Plasma bTMB algorithm 13 was applied to calculate pleural effusion-derived TMB for cfDNA in two groups, group A consisting of paired PE-F, and PE-E167 (n=23; P1-P7 & P16, P18-P32), group B consisting of paired PE-F and PE-FL (n=11; P4-P15). TMB calculated from PE-F were significantly higher than that calculated from either PE-E167 (Group A, median TMB for PE-F vs PE-E167, 17.8 mut/Mb vs 7.8 mut/Mb, p < 0.05) (Figure 3A) or PE-FL (Group B median TMB for PE-F vs PE-FL, 35.1 mut/Mb vs 7.8 mut/Mb, p < 0.05) (Figure 3B). Medium TMB from entire PE-F cfDNA samples in this study (20 mut/Mb) was much higher than TMB reported by Tong et al. (6.4 mut/Mb) as well as medium TMB value in NSCLC. TMB calculated from variants with VAF ≥ 0.1% also displayed significantly higher value in PE-F samples (Figure S1). These data suggested that the genetic information from long-fragment cfDNA in PE supernatant may interfere with TMB results.

### Distinct cfDNA profile, especially long-fragment cfDNA contained interfering variants affecting the accuracy of genetic testing

**Figure 4:**
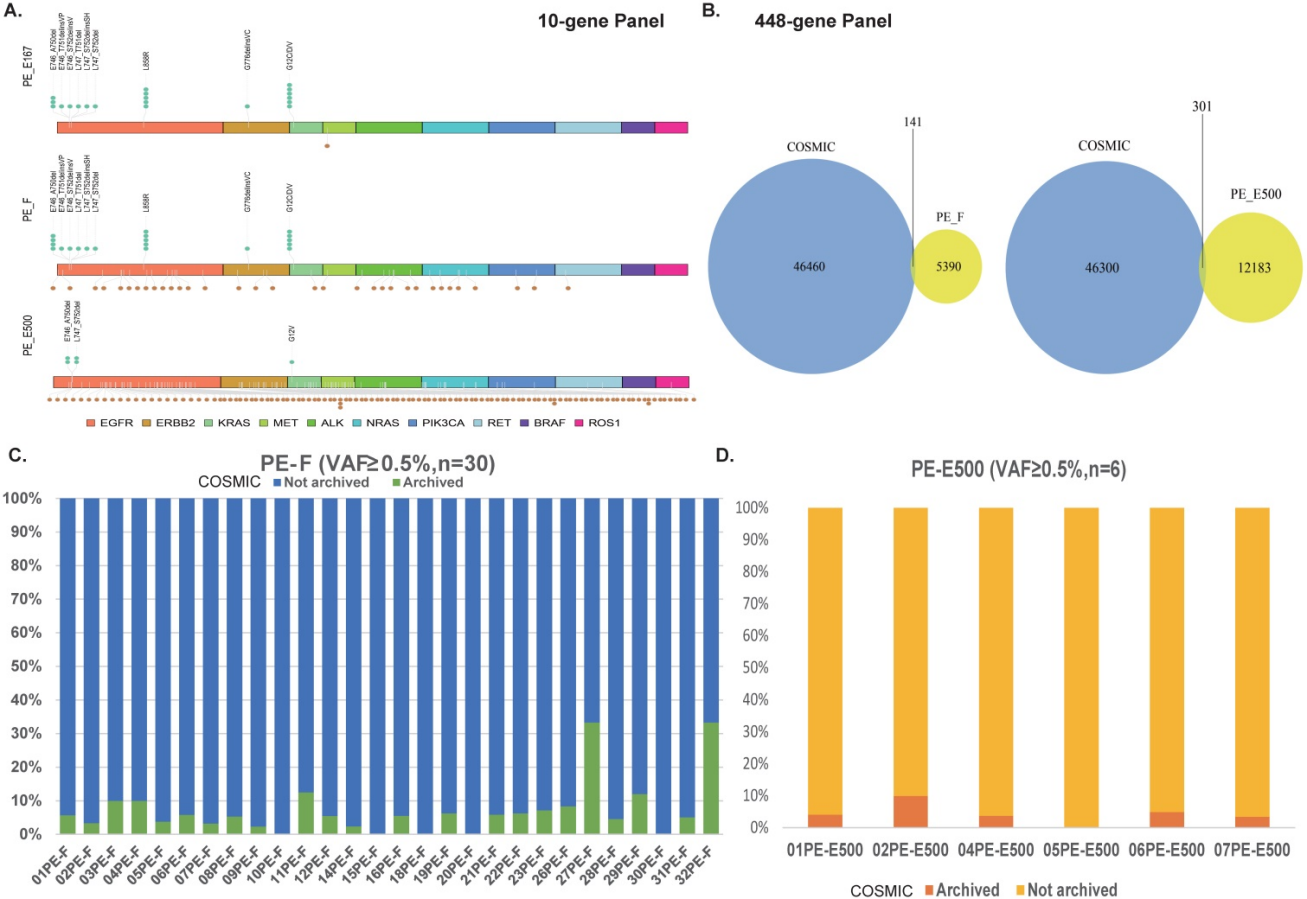
** Distinct cfDNA profile, especially long-fragment cfDNA in malignant pleural effusion contains COSMIC unreported variants. A.** An NGS 10-gene panel was used to compare the differences in the detected mutation sites between PE-E167, PE-F and PE-E500. 10 lung cancer-related driver genes were examined in PE-E167, PE-F and PE-E500. Hotspot variants are labeled green above each X-axis, unknown variations are labeled brown below each X-axis. **B.** Left panel: Venn graph displayed major variants with VAF ≥ 0.5% in PE-F samples were not reported and archived by COSMIC database. Right panel: Venn graph displayed major variants with VAF ≥ 0.5% in PE-E500 samples were not reported and archived by COSMIC database. **C.** Bar graph displayed distribution of COSMIC unarchived variants with VAF ≥ 0.5% in each PE-F sample. **D.** Bar graph displayed distribution of COSMIC unarchived variants with VAF ≥ 0.5% in six PE-E500 samples.

Thus, it was hypothesized that long fragments of cfDNA contained in PE-F might influence the results of genetic analysis. Variations in driver genes with VAF ≥ 0.5% was compared between PE-E167 and PE-F by NGS 10-gene panel. Paired PE-E500 samples were considered as the source of long-fragment cfDNA. Hotspot variations in drivers as marked by green dots above the X-axis were mainly observed in PE-167 group; While Unknown variations, marked with brown dots below the X-axis, were enriched in PE-F and PE-E500 samples (Figure 4A). Referring to the COSMIC database (Catalogue of Somatic Mutation in Cancer, https://cancer.sanger.ac.uk/cosmic), more than half of the variants in 10 NSCLC driver genes had not been reported (data not shown). Moreover, majority of the variants detected by comprehensive genomic profiling in PE-F and PE-E500 were not reported by COSMIC no matter with VAF ≥ 0.5% or 0.1% (Figure 4B, 4D, Figure S2A, S2D, and S2E). Especially, allele frequency of these COSMIC unarchived variants was majorly less than 1% in 30 PE-F samples or PE-E500 from 4 paired samples (Figure S2B, S2C).

Therefore, these unknown variations, majorly VAF < 1%, suspected to be interference introduced by long-fragment cfDNA, will affect accuracy of evaluating TMB if VAF ≥ 0.5% or 0.1% was set as cut-off value.

### Similar genetic profile was observed in paired PE-FL and PE-E167 cfDNA samples

PE-FL and PE-E167 cfDNA samples from two comparison groups yielded comparable TMB value. In addition, results from PE-FL and PE-E167 showed similar efficacy guiding tyrosine kinase inhibitor treatment as compared to result from clinical routine test (Figure S3).

**Figure 5:**
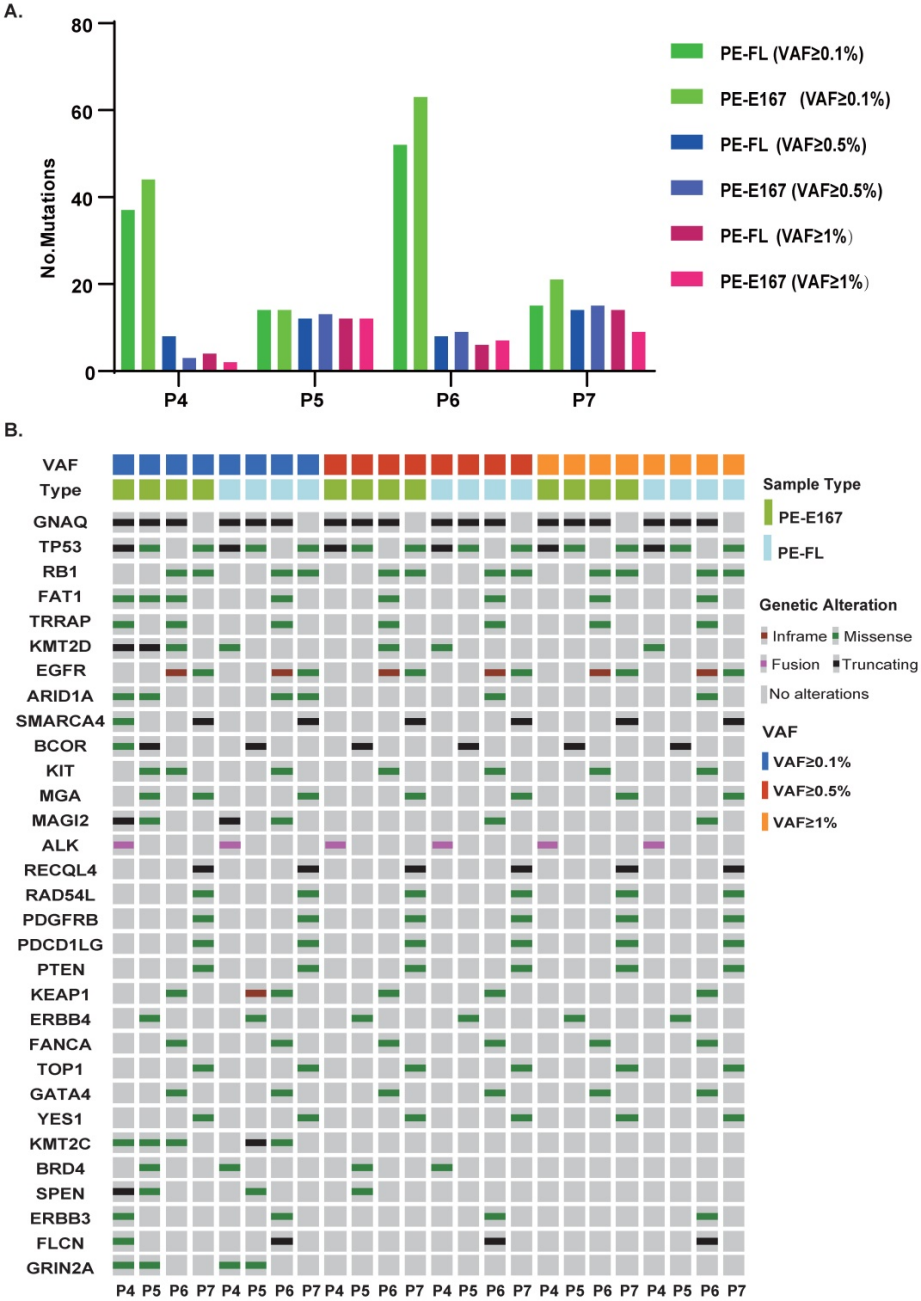
** Paired PE-E167 and PE-FL showed comparable genetic profile. A.** Bar plot shows distribution of detected mutation number under various VAF cut-offs in paired PE-E167 and PE-FL from four patients. **B.** Mutation profile under various VAF cut-offs in paired PE-E167 and PE-FL from four patients.

To clarify the concordance of genetic profile between these two cfDNA categories, paired PE-FL and PE-E167 were collected. Numbers of mutations were similar under three VAF cut-offs (0.1%, 0.5% and 1%) (Figure 5A). In parallel, PE-FL and PE-E167 displayed almost same profile of genetic mutations (Figure 5B). Therefore, it is recommended to use PE-FL to prepare sequencing library without the need for fragmentation of full-length cfDNA in clinical practice.

## Discussion

This study demonstrated at first time the distinct profile of cfDNA in malignant pleural effusion. This unique character, especially long cfDNA fragments in supernatant of pleural effusion showed potential impact on genetic analysis, especially TMB evaluation via introducing large number of interfering, non-cancer specific variants. Both use of total PE-cfDNA without fragmentation step and enriched PE-cfDNA at ~167 bp for NGS library preparation can avoid interference from long cfDNA fragments giving reliable and comparable results.

Pleural Effusion, as obtained by pleural drainage in clinics, besides liquid-based cytology (LBC) detecting cancer cells in patients with advanced cancer 14, 15 it has been previously proved to be suitable material for histological and genetic analysis 16-18. However, several obstacles including various numbers of tumor cells as well as leukocytes in PE sample, especially PE sediment, limited the sensitivity for detection of somatic variations. Thus, cfDNA in the supernatant of pleural effusion samples may be surrogate for molecular profiling. In this study, consistent with previous studies 5, 10, 19, PE supernatant cfDNA is a valuable surrogate material for detecting hotspot variations and TMB. Indeed, PE supernatant cfDNA was significantly superior to PE sediments (PE-S) in mutation detection. Thus, compared with PE sediment, PE supernatant cfDNA may be a better source of DNA without influence from other cell types like leukocyte, allowing genetic analysis available for patients with advanced lung cancer.

Genetic analysis of circulating cfDNA has been widely used in molecular diagnostics. DNA fragments within a specific range of size need to be isolated. Distribution of DNA fragments in PE supernatant has not been examined. Damaged tumor cells together with apoptotic or pyroptotic leukocytes may release large amounts of cfDNA in the pleural space, making pleural fluid a possible rich source of cfDNA. As Tong et al. reported, the amount of DNA was significantly higher in PE than in plasma. Therefore, analyzing and selecting cfDNA fragment in PE supernatant may help to improve the accuracy of genetic analysis. Our results have shown that longer DNA fragments in the PE supernatant cfDNA compared with plasma. This finding has not been reported so far. In lung cancer, the detection of hotspot mutations in driver genes such as *EGFR* was used as guidance for targeted therapy 20. On the other hand, the TMB value can be used to evaluate the efficacy of immunotherapy 21. Therefore, hotspot mutations and TMB were used as evaluative indicators to determine the influence from these long cfDNA fragments. No difference of driver gene mutations was observed between PE-F and PE-E167 or PE-FL by NGS lung cancer 10-gene panel or 448-cancer related gene panel. PE-F and PE-E500 detected more cancer-unrelated variations not archived in COSMIC, majorly with VAF ranging from 0.1%-1%, suggesting long-fragment cfDNA in PE supernatant may influence comprehensive genomic profiling. PE-F samples gave significantly higher TMB values which proved this concept.

To avoid interference from long fragments in PE-cfDNA, direct use of total PE-cfDNA without fragmentation step is recommended to construct the NGS library like PE-FL in this study as long fragments cannot be successfully captured and eventually excluded from the library. Another way is to enrich cfDNA fragments at ~167bp like PE-E167. Both of these two approaches can give trustable hotspot mutation result as well as reliable TMB evaluation. Enriching ~167bp cfDNA fragment may cost extra time, thus, directly using total PE-cfDNA without fragmentation step to prepare sequencing library is preferred in clinical practice.

This study has several limitations. First, the limited sample size might produce statistical bias. A larger study cohort for technical and clinical validation is warranted. Second, no paired tissue samples were available as “gold standard” for PE supernatant cfDNA tested result. Third, what is the potential biological mechanism for the generation of low-VAF non-tumor mutations has not been investigated in this study.

In conclusion, our data shown that long cfDNA fragments are more contained in PE supernatants than that in plasma cfDNA. Although PE-F can be used for detecting hotspot mutations, long fragments will affect the accuracy of test results, especially TMB by introduction of cancer-unrelated variants; especially with low allele frequency (0.1%-1%). Direct use of total PE-cfDNA without fragmentation step to construct sequencing library, instead of using fragmented full-length PE-cfDNA is recommended for clinical practice.

## Supplementary Material

Supplementary figures and tables.

## Figures and Tables

**Table 1:** Clinical characteristics of patients with advanced lung cancer

Clinical Character (n=32)	Number (%)
**Gender (%)**	
Female	11 (34.4)
Male	21 (65.6)
**Age (%)**	
<58	15 (46.9)
≥58	17 (53.1)
**Smoking history (%)**	
Current	13 (46.4)
Former	4 (14.3)
Never	11 (39.3)
**Clinical stage (%)**	
IVA	15 (46.9)
IVB	17 (53.1)
**T stage (%)**	
T1	8 (25.0)
T2	13 (40.6)
T3	4 (12.5)
T4	7 (21.9)
**N stage (%)**	
N1	1 (3.1)
N2	15 (46.9)
N3	16 (50.0)
**M stage (%)**	
M1a	9 (28.1)
M1b	6 (18.8)
M1c	17 (53.1)

## Abbreviations

bTMB: blood tumor mutational burden
cfDNA: cell free DNA
EGFR: epidermal growth factor receptor
GATK: Genome Analysis Toolkit
gDNA: genomic DNA
LBC: liquid-based cytology
MPE: malignant pleural effusion
NGS: next-generation sequencing
NSCLC: non-small cell lung cancer
PCR: polymerase chain reaction
PE: pleural effusion
PE-E167: PE cfDNA enriched to 167 bp
PE-E500: PE cfDNA fragment larger than 500 bp
PE-F: full-length cfDNA fragmented to 150-250 bp
PE-FL: full-length cfDNA from PE supernatant without fragmentation step during NGS library preparation
TMB: tumor mutational burden
VAF: variant allele frequency
