# Supplementary material for: Distinct profile of cell-free DNA in malignant pleural effusion of non-small cell lung cancer and its impact on clinical genetic testing
Source: Int J Med Sci. 2021 Jan 30;18(6):1510–8. doi: 10.7150/ijms.52306 (PMC7893565; doi:10.7150/ijms.52306)
Supplement: Supplementary file 1 — Supplementary figures and tables. [file ijmsv18p1510s1.pdf]

Supplementary Materials

Supplementary Figures

Supplementary Figure 1. TMB distribution calculated from variants with VAF ≥ 0.1% among various sample types.

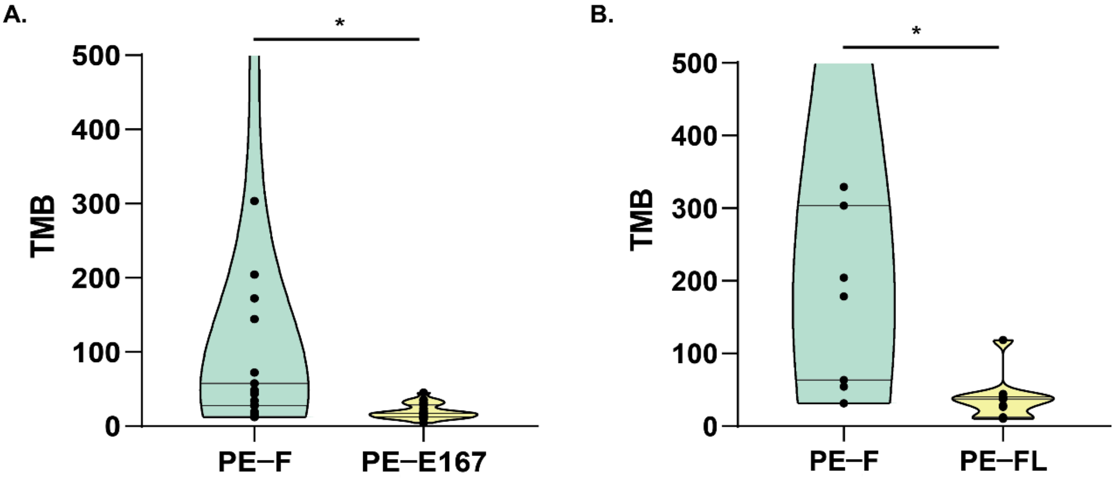

Supplementary Figure 2. Cancer unrelated variants with low allele frequency were dominant in PE-F and long fragment cfDNA.

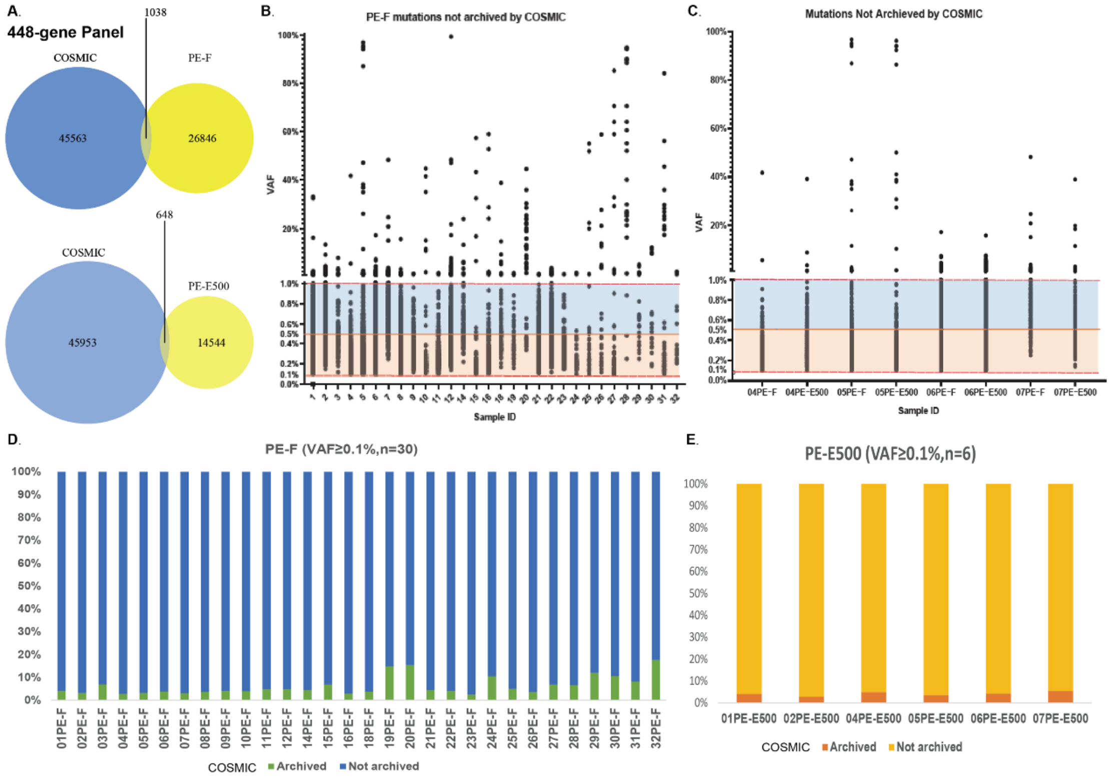

Supplementary Figure 3. Kaplan Meier progression-free survival curve for patients that have EGFR sensitive mutants, ALK or ROS1 fusion detected from PE-E167, PE-FL or clinical routine test.

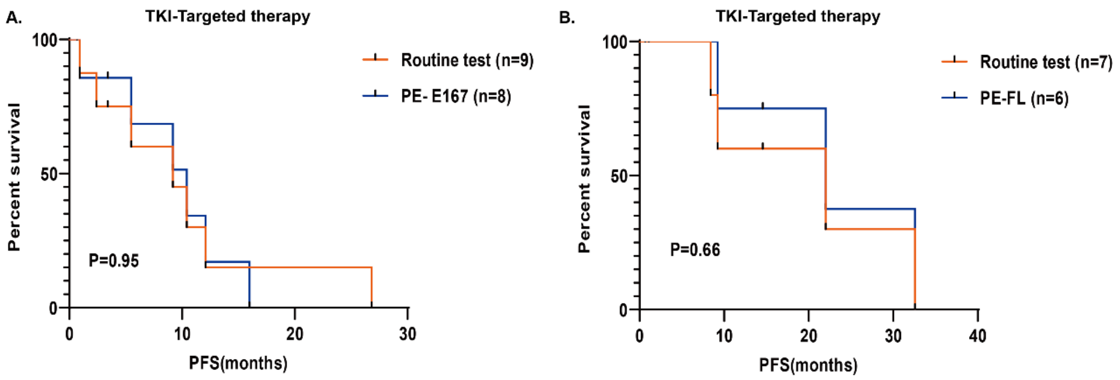

Supplementary Table 1. Genes detected by the 448-gene panel

ABCB1	*BRD4*	*CUL3*	*FAS*	*H3F3C*	*KMT2D*	*MYD88*	*PIK3CG*	*RET*	*STAT4*
ABL1	*BRIP1*	*CYLD*	*FAT1*	*HAVCR2*	*KRAS*	*NBN*	*PIK3R1*	*RHEB*	*STK11*
ABL2	*BTG1*	*CYP2D6*	*FBXW7*	*HGF*	*LAG3*	*NCOA3*	*PIK3R2*	*RHOA*	*SUFU*
ACVR1B	*BTK*	*DAXX*	*FCGR2B*	*HIST1H1C*	*LATS1*	*NCOR1*	*PIM1*	*RICTOR*	*SUZ12*
AKT1	*C11orf30*	*DCUN1D1*	*FGF10*	*HIST1H2BD*	*LATS2*	*NF1*	*PLCG2*	*RIPK4*	*SYK*
AKT2	*CALR*	*DDR1*	*FGF14*	*HIST1H3B*	*LCK*	*NF2*	*PLK2*	*RIT1*	*TAF1*
AKT3	*CARD11*	*DDR2*	*FGF19*	*HLA-A*	*LMO1*	*NFE2L2*	*PMS1*	*RNF43*	*TAOK1*
ALK	*CASP8*	*DICER1*	*FGF23*	*HNF1A*	*LRP1B*	*NFKBIA*	*PMS2*	*ROS1*	*TBX3*
ALOX12B	*CBFB*	*DIS3*	*FGF3*	*HRAS*	*LYN*	*NKX2-1*	*PNRC1*	*RPS6KB1*	*TCF7L1*
AMER1	*CBL*	*DNMT1*	*FGF4*	*HSD3B1*	*LZTR1*	*NOTCH1*	*POLD1*	*RPS6KB2*	*TCF7L2*
APC	*CCND1*	*DNMT3A*	*FGF6*	*HSP90AA1*	*MAGI2*	*NOTCH2*	*POLE*	*RPTOR*	*TERC*
AR	*CCND2*	*DOT1L*	*FGF7*	*ICOS*	*MAP2K1*	*NOTCH3*	*POLE4*	*RUNX1*	*TERT*

ARAF	*CCND3*	*EGFR*	*FGFR1*	*ICOSLG*	*MAP2K2*	*NOTCH4*	*PPARG*	*RUNX1T1*	*TET1*
ARFRP1	*CCNE1*	*EIF4A2*	*FGFR2*	*IDH1*	*MAP2K4*	*NPM1*	*PPP2R1A*	*SCN8A*	*TET2*
ARID1A	*CD274*	*EP300*	*FGFR3*	*IDH2*	*MAP3K1*	*NRAS*	*PRDM1*	*SDHA*	*TGFBR2*
ARID1B	*CD79A*	*EPCAM*	*FGFR4*	*IGF1*	*MAP3K13*	*NSD1*	*PREX2*	*SDHAF2*	*TIGIT*
ARID2	*CD79B*	*EPHA3*	*FH*	*IGF1R*	*MAP3K14*	*NTRK1*	*PRKAA1*	*SDHB*	*TMEM127*
ARID5B	*CD80*	*EPHA5*	*FLCN*	*IGF2*	*MAPK1*	*NTRK2*	*PRKACA*	*SDHC*	*TMPRSS2*
ASXL1	*CD86*	*EPHA6*	*FLT1*	*IKBKE*	*MAPK3*	*NTRK3*	*PRKAR1A*	*SDHD*	*TNFAIP3*
ATM	*CDC73*	*EPHA7*	*FLT3*	*IKZF1*	*MAPK4*	*NUP93*	*PRKCI*	*SETBP1*	*TNFRSF14*
ATR	*CDH1*	*EPHB1*	*FLT4*	*IL7R*	*MAX*	*OXSR1*	*PRKDC*	*SETD2*	*TNFRSF18*
ATRX	*CDK12*	*ERBB2*	*FOXA1*	*INHBA*	*MCL1*	*PAK1*	*PRSS8*	*SF3B1*	*TNFRSF9*
AURKA	*CDK4*	*ERBB3*	*FOXL2*	*INPP4A*	*MDC1*	*PAK3*	*PTCH1*	*SIK1*	*TOP1*
AURKB	*CDK6*	*ERBB4*	*FOXO1*	*INPP4B*	*MDM2*	*PAK7*	*PTEN*	*SKP2*	*TOP2A*
AXIN1	*CDK8*	*ERCC1*	*FOXP1*	*INSR*	*MDM4*	*PALB2*	*PTPN11*	*SLIT2*	*TP53*

AXIN2	*CDKN1A*	*ERCC2*	*FRS2*	*IP6K1*	*MED12*	*PAPPA2*	*PTPRD*	*SMAD2*	*TRAF7*
AXL	*CDKN1B*	*ERCC3*	*FUBP1*	*IRF2*	*MEF2B*	*PARK2*	*PXDNL*	*SMAD3*	*TRRAP*
B2M	*CDKN2A*	*ERG*	*FYN*	*IRF4*	*MEN1*	*PARP1*	*QKI*	*SMAD4*	*TSC1*
BAP1	*CDKN2B*	*ERRFI1*	*GABRA6*	*IRS2*	*MET*	*PAX5*	*RAC1*	*SMARCA4*	*TSC2*
BARD1	*CDKN2C*	*ESR1*	*GATA1*	*JAK1*	*MGA*	*PAX8*	*RAD21*	*SMARCB1*	*TSHR*
BCL2	*CEBPA*	*ETV1*	*GATA2*	*JAK2*	*MITF*	*PBRM1*	*RAD50*	*SMARCD1*	*U2AF1*
BCL2L1	*CHD2*	*ETV4*	*GATA3*	*JAK3*	*MLH1*	*PDCD1*	*RAD51*	*SMO*	*VEGFA*
BCL2L11	*CHD4*	*ETV5*	*GATA4*	*JUN*	*MLH3*	*PDCD1LG2*	*RAD51B*	*SNCAIP*	*VHL*
BCL2L2	*CHEK1*	*ETV6*	*GATA6*	*KDM5A*	*MPL*	*PDGFRA*	*RAD51C*	*SOCS1*	*WISP3*
BCL6	*CHEK2*	*EZH2*	*GLI1*	*KDM5C*	*MRE11A*	*PDGFRB*	*RAD51D*	*SOX10*	*WT1*
BCOR	*CIC*	*FAM175A*	*GNA11*	*KDM6A*	*MSH2*	*PDK1*	*RAD52*	*SOX17*	*XPC*
BCORL1	*CREBBP*	*FAM46C*	*GNA13*	*KDR*	*MSH3*	*PDPK1*	*RAD54L*	*SOX2*	*XPO1*
BCR	*CRKL*	*FANCA*	*GNAQ*	*KEAP1*	*MSH6*	*PGR*	*RAF1*	*SOX9*	*XRCC1*

BIRC3	*CRLF2*	*FANCC*	*GNAS*	*KEL*	*MST1R*	*PHF6*	*RANBP2*	*SPEN*	*YES1*
BLM	*CSF1R*	*FANCD2*	*GREM1*	*KIT*	*MTOR*	*PIK3C2B*	*RARA*	*SPOP*	*ZBTB2*
BMPR1A	*CSF3R*	*FANCE*	*GRIN2A*	*KLF4*	*MUTYH*	*PIK3C2G*	*RASA1*	*SPTA1*	*ZNF217*
BRAF	*CTCF*	*FANCF*	*GRM3*	*KLHL6*	*MYB*	*PIK3C3*	*RB1*	*SRC*	*ZNF703*
BRCA1	*CTLA4*	*FANCG*	*GSK3B*	*KMT2A*	*MYC*	*PIK3CA*	*RBM10*	*SRSF2*	*ZRSR2*
BRCA2	*CTNNA1*	*FANCI*	*GSTP1*	*KMT2B*	*MYCL*	*PIK3CB*	*RECQL4*	*STAG2*	
BRD3	*CTNNB1*	*FANCL*	*H3F3A*	*KMT2C*	*MYCN*	*PIK3CD*	*REL*	*STAT3*	

Supplementary Table 2. Genes detected by the 10-gene panel

ALK
BRAF
EGFR
ERBB2
KRAS
MET
NARS
PIK3CA
RET
ROS1
